# Supplementary material for: Yiai Fuzheng decoction inhibits triple-negative breast cancer by remodeling the immune microenvironment
Source: Front Immunol. 2025 Sep 30;16:1615631. doi: 10.3389/fimmu.2025.1615631 (PMC12518410; doi:10.3389/fimmu.2025.1615631)
Supplement: Supplementary file 2 [file DataSheet1.zip › Supplementary File 2/Treatment/C_vs_D/01_Basic_Statistics/Metabolite_Class_Statistics/Class_Pieplot.pdf]

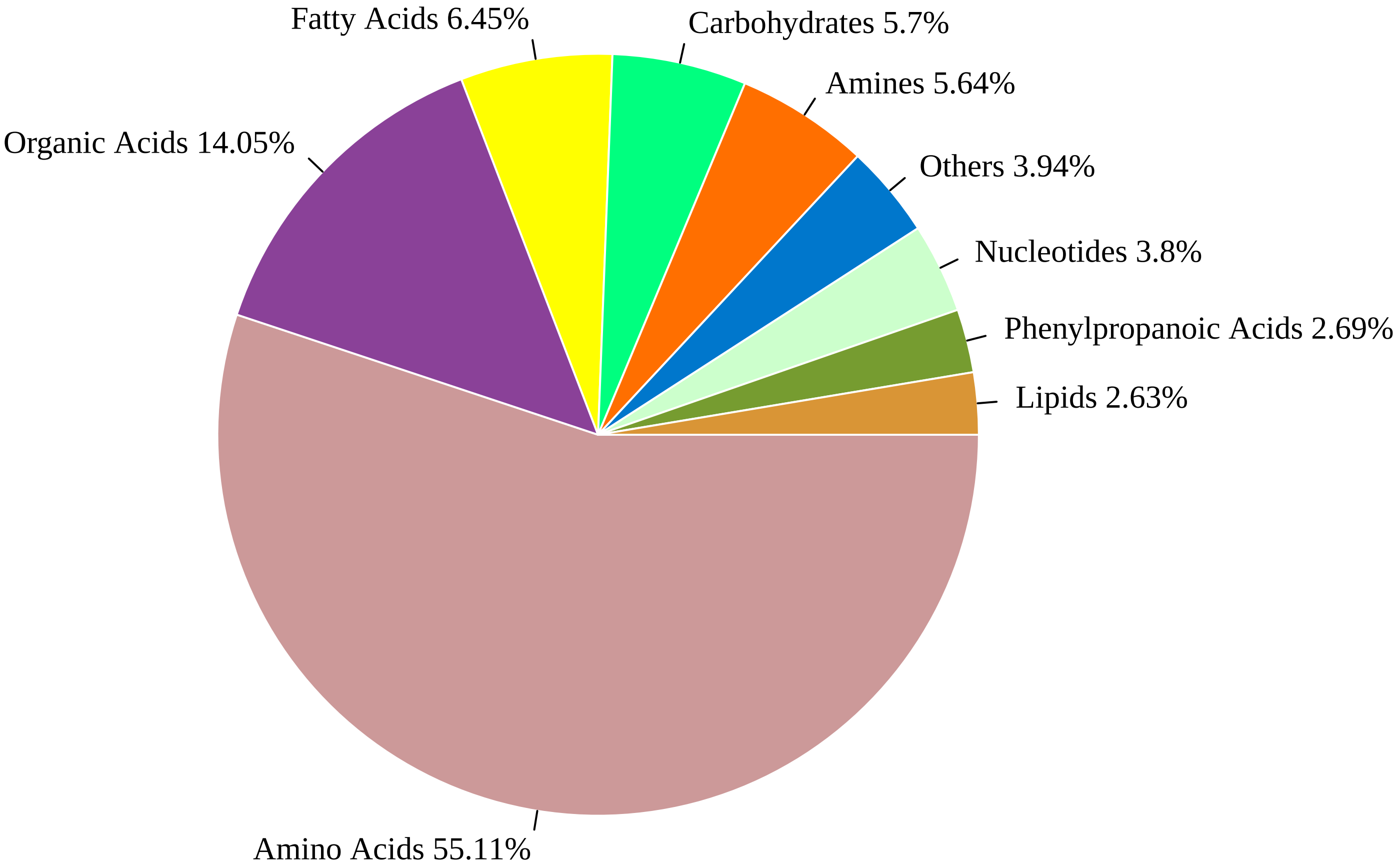

Fatty Acids 6.45%
Carbohydrates 5.7%
Amines 5.64%
Organic Acids 14.05%
Others 3.94%
Nucleotides 3.8%
Phenylpropanoic Acids 2.69%
Lipids 2.63%
Amino Acids 55.11%
